# Editorial: The necessity of adult learning methods in programs of intensive study

**DOI:** 10.1155/S146392469100024X

**Published:** 1991

**Authors:** Mary E. Ferriter

**Affiliations:** Zymark Corporation Zymark Center Hopkinton Massachusetts 01748 USA

## Abstract

This article presents the educational methodologies that prove effective in adult educational programmes of intensive study. The many facets of a quality educational programme are discussed and I will focus on four topics that any adult educational programme must have: an adult learner, an instructor of adults, a curriculum, and a response to outside forces.

These topics become increasingly critical when one examines the components of technical education and, especially, an intensive training programme in laboratory automation systems. The adult will be discussed as a learner and the associated myths and principles. Next, I will focus on the instructor and his/her necessary personal and professional qualities, including essential skills and psychological elements required. Aspects of curriculum will then be studied. The conventional and the innovative approaches to curriculum design, development, and delivery differ markedly. Development and delivery are so closely linked to the curriculum that both will be discussed under the one title of ‘curriculum’. The final discussion will focus on the outside forces that directly and indirectly affect adult education; since these are many, they are limited to a few salient ones.


Journal of Automatic Chemistry, Vol. 13, No. 4 (July–August 1991), pp. 123–128

### Editorial: The necessity of adult learning methods in programs of intensive study

**Mary E. Ferriter**

*Zymark Corporation, Zymark Center, Hopkinton, Massachusetts 01748, USA*

*This article presents the educational methodologies that prove effective in adult educational programmes of intensive study. The many facets of a quality educational programme are discussed and I will focus on four topics that any adult educational programme must have: an adult learner, an instructor of adults, a curriculum, and a response to outside forces.*

*These topics become increasingly critical when one examines the components of technical education and, especially, an intensive training programme in laboratory automation systems. The adult will be discussed as a learner and the associated myths and principles. Next, I will focus on the instructor and his/her necessary personal and professional qualities, including essential skills and psychological elements required. Aspects of curriculum will then be studied. The conventional and the innovative approaches to curriculum design, development, and delivery differ markedly. Development and delivery are so closely linked to the curriculum that both will be discussed under the one title of 'curriculum'. The final discussion will focus on the outside forces that directly and indirectly affect adult education; since these are many, they are limited to a few salient ones.*

A strong commitment to education is necessary in an economy stressing technological development and a competitive edge in a society with an increasing knowledge base [1]. Estimates predict that within the next decade, the 10 top US industries will create 9·6 M new jobs [2]. Industry will move toward a higher technology focus, and manufacturing will experience parallel changes as a result. Therefore, the industries will require workers familiar with high-technology systems. Within this classification lie all varieties of laboratory automation systems. Consequently, education of the work force becomes critical as does the necessity to examine worker readiness to receive education and accept flexible roles.

Marie Kraska, the Field Editor at Mississippi State University, summarized the need for enhanced technical education:

> as we approach the 1990s, technical education will assume a major role in preparing tomorrow's work force. Technological innovations hold implications not only for the work place, but for other facets of life as well. Changing lifestyles, the shifting roles of women and men, the trend toward service industries, the need for worker retraining and cross-training, and the trend toward self-help, and worker satisfaction will influence technical education to provide state-of-the-art training and job preparation.

Although feminine gender has been chosen in this text for the sake of convenience, I intend that it should represent both females and males equally.

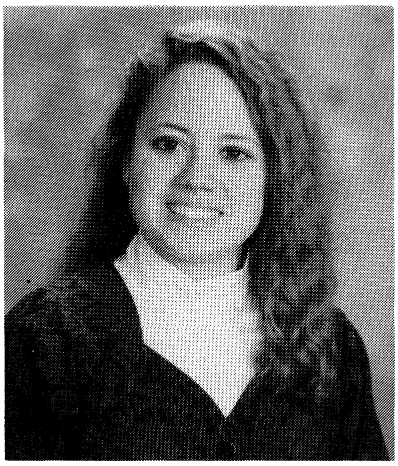

***Mary E. Ferriter joined Zymark Corporation in 1990 as a Technical Training Specialist. She has earned an M.Ed. in Adult Education from Worcester College of Education and a B.A. in Biochemistry as well as Learning Psychology from Dartmouth College. This science and education combination is rare.***

***Mary's background is in training styles, curriculum development, and participatory-learning methods. She specializes in programmes of intensive study. Her educational experience together with her innovative programme design and her knowledge of the adult learner have helped to keep Zymark's training programmes in parallel with the educational strategies of the decade.***

### The adult as learner

When dealing with adults, an instructor should remember three key points:

(1) Adults learn what they are ready to learn – they will learn only those ideas and concepts perceived as being new to them [3].

(2) Adults come to learn for practical reasons – they need to apply concepts today [4].

(3) Adult learners dislike having their time wasted – they require well-organized, efficiently managed, and productive programmes [4].

All adult learners are different, since each is a unique individual. However, at the base of adult learning are some characteristics, myths, and principles that instructors of adults should keep in mind. All become particularly important when discussing technical training and are even more important when intensive technical training is involved (see figure 1).

0142–0453/91 $3.00 © 1991 Taylor & Francis Ltd.

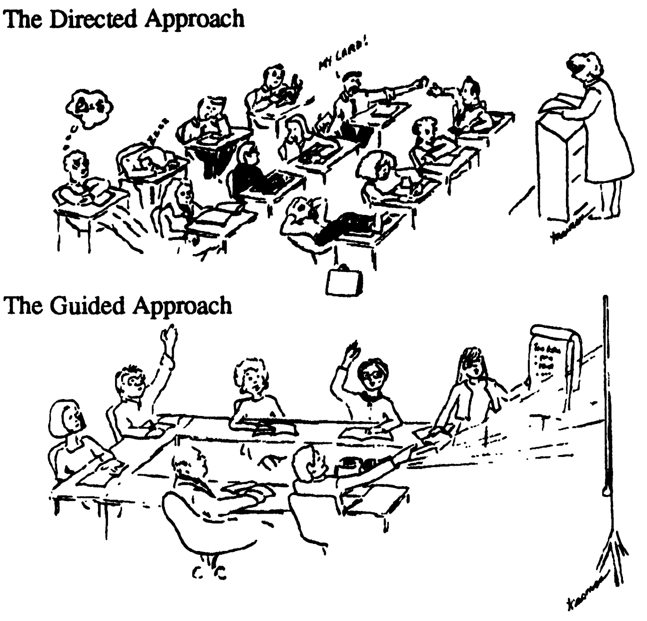

*Figure 1. Issues of the learner in andrological and pedalogical models.*

At the root of adult education are the words andragogy and pedagogy; the former meaning the art of helping adults to learn, the latter meaning the art of teaching children. Adults need a guided approach; children need a more directed approach. The andragogical and pedagogical models of education incorporate different considerations as illustrated in table 1. The instructor must remember and respect that adults come to class with a whole array of information gained from past experiences. Even if the subject is completely unfamiliar, the instructor can structure the curriculum around these inherent thought processes such that adult students do not feel inhibited, afraid, or dominated. Adults can think critically and solve problems. By their own intellectual processes, adult learners can guide their learning through discussions and processing based on their intellectual development.

In allowing for past experiences and an andragogical educational perspective, the instructor can encourage adult learners to use their own resources, and can, thus, present information in a new refreshing manner.

### The instructor of adults

Knox [5] states that most instructors 'in adult educational programmes are expert in the content they teach, but they usually have little preparation in the process of helping adults learn'. This phrase is the essence of andragogy. If one keeps in mind that andragogy is the art of helping adults to learn, then anyone involved with programme design, development, and delivery must keep this meaning as the central most focus. Key to all of these components is the instructor.

Those who help adults learn have several different titles, among them facilitator, adult educator, trainer, teacher, and instructor. In technical training I prefer the word instructor, for the word implies a person who lays the groundwork of guidance, offers a variety of presentational methods, and develops theory into practical course application. The qualities of a model instructor are multifold: from curriculum-planning skills to curriculum-delivery skills to interpersonal skills. The qualities of an instructor establish the backbone for the adult-learning process.

Educational delivery is, at its base, contingent on the instructor's own behaviour, as well as the instructor's awareness of her students' individual and group behaviours [4]. Every instructor brings with her a set of values, norms, and expectations underlying the paradigms of instruction. Each student becomes influenced by what the instructor believes and how she behaves. This sometimes ignored influence will determine student motivation, self-regard, attention toward and acquisition of content, dependency on the instructor for answers, as well as overall attitudes concerning the group, subject, and instructor [4].

Interpersonal skills are tightly interwoven in the lattice of education [6]. The initial establishment of the educational climate sets the tone for subsequent learning. The psychological climate must be open, flexible, friendly, and mutually supportive [7]. Instructor mannerisms interpreted as threatening or condescending will prove hazardous to the student's future learning and trust, as well as information interchange.

Other psychological elements relate to more cognitively based elements. Miller and Dollard [8, 9] isolated three components of teaching – cues, engagement, and reinforcement – which, when used appropriately, have an enormous effect on learning [10].

Cues can be used variously in education. They are used to show material to be learned and to provide guidance in how to learn it. Some examples of cues would be: (1) the delivery of education in a format that relates new material to previous experiences or learning: a connection to or bridging of the old material to the new; (2) the use of a precourse-knowledge assessment as a means to prime students to the salient points of material to be covered; (3) the use of appropriately phrased questions that build on the already existent knowlege base and that are redundant to those questions used on the precourse-knowledge assessment; (4) the use of a structured curriculum presented in a logical sequence by which the instructor shows a general overview of the whole and builds parts upon parts until a comprehensive understanding is achieved. Instructors should reveal their goals and objectives at the outset while taking into account the students' personal goals and objectives. Clarity is crucial at the start. In intensive training sessions, the key is a concentration of learning by use of a time-efficient delivery method. Extraneous material must be eliminated.

Engagement in educational delivery involves instructor expectation. Although an instructor must be able to set realistic expectations to a degree that motivates students to prolonged perseverance, to extended efforts toward learning, and to newer and higher plateaus.

*Table 1. Issues of the learner in andrological models.*

| Learner's issues | Andragogical considerations | Pedagogical considerations |
|---|---|---|
| Self-concept | Self-directed | Dependent personality |
| Experience | Rich resource for learning by self and others | Insufficient, needs to be built on |
| Readiness to learn | Develops from life tasks and problems | Uniform by age level and curriculum |
| Orientation to learning | Task or problem centered | Subject centered |
| Motivation | Internal incentives, curiosity | External rewards and punishments |

Engagement also involves instructor questioning. The instructor should consider the appropriate time lapse from the end of her question to the beginning of the learner's answer. An instructor waits, on average, only one second before answering a question herself [11]. She needs to place emphasis on pausing for at least 15 seconds while waiting for an answer. The use of adjunct questioning with appropriate timing will prompt the students to formulate their own answers using their problem-solving skills and their previous knowledge. This approach generally leads to more thought-provoking answers.

Encouragement, incentives, and student morale typify good group-learning process. However, the main emphasis needs to be on student progress and should avoid rewards. Homework and feedback thereby become important correctives and reinforcements. Homework extends the learning time, encourages comprehension of material, and promotes reflective integration of the material into the learner's lifestyle. Homework for adults must focus on practicality, and, most important, must help the learner derive practice from theory. Even the word homework may require alteration. The word can bring with it some negative connotations, perhaps established during high-school experiences. Such words as 'problem sets', 'knowledge exercises', or 'case studies' connote more open expectations and, at the same time, stress more practical aspects of learning.

Challenge is at the base of an instructor's quality. The instructor should be challenged by the course content and be able to assess the students so that she can present the course content in a challenging and fulfilling manner. Education should challenge all participants. A challenging curriculum enhances student reflection, motivation, and comprehension. Furthermore, challenge is fundamental to critical thinking [12].

### The curriculum

Curriculum development constitutes the structure of any educational programme. A curriculum consists of at least four components:

(1) Needs assessment.

(2) Learning objectives.

(3) Instructional strategies.

(4) Evaluation methods.

Within each component are materials elemental to the programme's success. Organization and time commitment are mandatory.

At the forefront is needs assessment. The term indicates a type of survey, questioning, or determination that will set the precedent for curriculum content. The complete needs assessment will reveal any gaps between information known and knowledge to be gained [13]. This assessment should also take into account what the learner expects to achieve by attending the course. There is no consensus on the most appropriate method to use in needs assessment [14]. Thus, when designing the needs assessment, one should keep in mind the desired purposes and goals of the course as well as the educational needs of the students [15]. A needs assessment can entail any or all of the following: field observations, surveys, interviews, record checks, task analyses, assessment sessions, performance determinations. When gathering data, one can frame questions concerning what information the learners desire, what skills they need, and what problems exist.

In formulating learning objectives, one must keep in mind three factors. First, each objective must include action verbs. Connotatively and denotatively, action verbs express performance: 'to draw', 'to write', 'to discuss', and 'to demonstrate' exemplify action verbs. Second, each objective should include a situation, an action, and a means to evaluate the action. Third, each objective should display realism. Objectives must parallel the participants' abilities, expected goals, and desired proficiencies [16]. An example is illustrated below:

> Following the section on robotic command language, the participant will write a detailed program which will incorporate at least four different commands and which will perform the desired action upon execution.

In the above example, the robotic command language indicates the situation, the action verb 'to write' was chosen, and the evaluation will be gained through the student's use of the four different commands as well as through successful execution of the program. Above all else, objectives should centre around active learning, practical learning, participative learning.

Commentary concerning instructional strategies in andragogical settings could encompass a textbook of information. I will briefly review lecture, discussion, demonstration, and participatory learning.

Lecture should be used sparingly. Lecture should be placed carefully and critically within the curriculum and should never exceed 15 minutes at any given time. Lecture is by far the easiest method of educational delivery. It requries the smallest amount of preparation time, allows the largest degree of instructor control, and is the most traditional, therefore the most comfortable, means of presentation. Instructors should avoid pure lecture format, since learning retentions from lecture-formatted material is low.

Discussion usually follows a lecture or an audio-visual presentation of slides, films, or charts. Discussion can occur in a group that encompasses the entire class, in small or large groups, or in groups that leave the classroom and rejoin for a wrap-up session. Every discussion session requries a facilitator to keep the conversation oriented. The facilitator's responsibilities normally involve starting the discussion through openended questions, building the discussion on any previous knowledge, and reaching conclusions through a goal-centred approach.

Demonstrations are useful in technical training. They are customarily used to show the physical elements and attributes of procedures. Usually instructor led, demonstrations can hide or mask many of the important details. Demonstration can serve as an excellent introductory method to participatory learning.

Participatory learning is the goal of adult education [17, 18]. It can involve many elements, such as simulations, role playing, written exercises, or problem sets. Participatory learning requires an enormous amount of preparatory time, materials coordination, and educational delivery time. For these reasons and more, instructors tend to avoid it. However, participatory learning represents the key building block to a complete integration and comprehension of information. It can be used anywhere on any topic. This idea is difficult for instructors to grasp. Creativity, innovation, and initiative are *all* required in using participatory learning, especially in situations where participatory learning has never been tried. Anything new, nontraditional, and variable involves risk. The amount of risk opted by individual instructors seems correlated to the instructor's identity, self-worth, and outlook on life. The most successful curriculums reflect the energy of the developer, her devotion to education, and her personal character. When participatory learning is exercised, any potential for failure becomes counterbalanced by the learners' motivation, comprehension, and retention rates.

An exceptional curriculum will alternate between all four of these training methods. Because the average adult attention span lasts 17 minutes, the mode of presentation must change every 10 to 15 minutes. By 'change', I do not mean radically or abruptly. For example, since most instructors and participants perceive a lecture format as comfortable, lecture can occupy the first minutes of a class. Lecture can lead into a slide presentation with accompanying commentary, which can proceed to small discussion groups, back to a class summary, and into a flipchart presentation of the important points for focus. From here a demonstration can be given followed by a participatory learning session. Already, 90 minutes could have passed, the participants would have been mentally and physically stimulated and are unlikely to have experienced boredom. They will, furthermore, have had an opportunity to get up and walk to and from their discussion groups. As long as the basic form of presentation is changed every 15 minutes, participants' attention spans should never be exceeded.

In general, instructors try to present too much material into too short a period of time. Because content is familiar to the instructor, she can overlook the learner's comprehension for the sake of completeness. The learner is new to this subject matter and does not and cannot grasp the information as quickly or as readily as the instructor. When the learner reaches overload, she will become incapable of processing any more, her mind will shut down, and she will no longer be able to focus on the learning. Instructors must present the material in manageable chunks. The preliminary needs assessment can help determine the size of the chunks based upon the students' backgrounds and characteristics.

Since each student brings with her a preference toward a certain teaching style, it becomes crucially important to have variety within the curriculum. Table 2 regroups the four major presentational skills into sensory groupings. Adults learn best from multisensory presentational approaches. Such approaches require great expenditures of preparation time, an ability to be flexible in course presentation, an aptitude to recognize when a particular approach is not working, and the flexibility, knowledge, and aptitude to change the presentational format when necessary.

Programme closure, programme review, and potential programme redesign or restructure rely on the evaluations. Evaluations of programmes can take many forms, depending upon the desired end result. The evaluation must be formulated around the programme components that information is desired: needs analysis, education objectives, learning activities, students, written materials, physical surroundings, and so forth [19]. Written surveys, questionnaires, or interviews can be used for assessment. Such evaluation means as quizzes, homework assignments, or projects can yield information about the learner's educational achievement. Since evaluation can measure the success of the training, the evaluation results provide the instructor feedback and data for improvement. Evaluative feedback helps instructors keep the course objectives and goals on track. A student's learning motivation is nurtured through evaluative feedback.

### Outside forces

Many environmental, physical, and societal influences can affect the learning process. The room or building where education takes place has many characteristics and details that require attention when adult learners are involved. Adults are sensitive to their environments [4]. Good temperature control and adequate lighting are necessities. The chairs should be comfortable, and sufficient desk or table space must be provided. The seating and view are also critical elements. Seating is best set up in a U-, a circular-, or a box-shaped arrangement. This allows each student to see others' faces. All participants must have a clear view of the instructional area, since obstructed views prove detrimental to student motivation, attention, and involvement. Presentations also should be easily heard from all parts of the room. This may entail raised speakers so that the sound is not muffled by the carpet, floor, or anything else. For visual concerns, slides, overheads, and charts all need to be sufficiently large for easy viewing. Distracting sounds from outside or inside the classroom can prove detrimental to learning, and the instructor should try to control this type of distraction. The physical environment must encourage participation.

Societal influences reign strongly over adults as learners. Widely held societal attitudes become stronger as the adult ages. For instance, some common assumptions are that all adults learn alike, that old dogs cannot be taught new tricks, that education is for young people, and that adults will exert as little time and energy as is possible. Each of these is a myth which practice has disproved.

Adults exert their own forms of outside forces on learning. With this statement I mean that adults generally work during the day and then attend educational programme during the evening, or *vice versa*. Adults have a home life and social activities that are sometimes put aside during their educational endeavours. Instructors must be mindful of these issues and make associated allowances. Adults may sometimes be late: traffic may have been heavy, a child may be sick, childcare may have been late, or work may have run over. Adults may sometimes leave early for similar reasons. These same issues can also contribute to fatigue, lethargy, or lack of attention. All these and other possible drains require instructor consideration in adult education.

### Conclusion

Effective and well-prepared training programmes require careful design, development, and delivery. This is especially true with intensive training programmes. Programme sponsors must consider aspects of learner needs, instructor qualities, curriculum composition, and societal influences. All of these factors set the direction for the programme flow and student interest.

The individual natures of adult learners provide them with the motivation and desire to learn. However, each learner arrives at class with a variety of life experiences, knowledge bases, and learning preferences. This *potpourri* of characteristics requries instructor aptitude.

An apt and able instructor will assess the learner's needs, analyse the desired proficiencies, and present an appropriate curriculum. Instructors must possess a multitude

*Table 2. Multisensory categorization of presentational skills and strategies.*

| Visual | Auditory | Kinesthetic |
|---|---|---|
| Boards | Audiotapes | Actual Items |
| Charts, graphs, diagrams | Audiovisuals | Case histories and studies |
| Computer-aided instruction | Discussions | Computer aided-instruction |
| Demonstrations | Films | Flowcharting |
| Films | Games | Games |
| Handouts | Group projects | Individual student models |
| Models | Lectures | On-the-job practice |
| Overhead projectors | Panel discussion | Paper and pencil tests |
| **Participatory learning** | **Participatory learning** | **Participatory learning** |
| Programmed instruction | Question-and-answer sessions | Programmed instruction |
| Reference materials | | Role playing |
| Slides | | Simulations |
| Training manuals | | |
| Written examples | | |

of instructional strategies. With adult learners, a multisensory and participatory learning environment promotes motivation, increases comprehension, and amplifies knowledge retention.

The goal of any intensive training programme is application, practicality, and retention of knowledge.

### Acknowledgements

I wish to thank Joseph Giroux for his technical assistance and Joanne Morris for designing the tables. Special thanks goes to Lynn Nasman, the artist responsible for adding humour to my article.

### References

1. Mohamed, D. A., *The Vocation Education Journal,* **63** (August 1988), 49(1).
2. Spikes, W. F., *Adult Learning,* **1** (September 1989), 10(1).
3. Karmos, J. S. and Greathouse, L. *Vocational Education Journal,* **64** (April 1989), 28(2).
4. Knowles, M. S. and Associates, *Applying Modern Principles of Adult Learning* (Jossey-Bass, San Francisco, 1984).
5. Knox, A., **Helping Adults Learn** (Jossey-Bass, San Francisco, 1986).
6. Galbraith, M. W., *Lifelong Learning: An Omnibus of Practice and Research,* **12** (1989), 10(6).
7. Draves, W., *How to Teach Adults* (LERN, Manhattan, 1984).
8. Miller N. and Dollard, J., *Social Learning and Imitation* (Yale University Press, New Haven, Connecticut, 1941).
9. Dollard, J. and Miller, N., *Personality and Psychotherapy* (McGraw-Hill, New York, 1950).
10. Walberg, H. J., *Handbook of Research on Teaching,* Ed. Wittrock, M. C. (Macmillan, New York, 1986).
11. Wal-berg, H. J., *Phi Delta Kappan,* **71** (February 1990), 470(9).
12. Egan, G., *The Skilled Helper* (3rd edn.), (Brooks/Cole, Monterey, 1986).
13. Cameron,. C., *Lifelong Learning: An Omnibus of Practice and Research,* **11** (January 1988), 25(4).
14. Nowlen, P., *Developing, Administering, and Evaluating Adult Education,* Eds. Knox, A. and Associates (Jossey-Bass, San Francisco, 1980).
15. Stinard, T., *Chicago Performance and Instruction,* **5** (1986), 9(2).
16. Galbraith, M. W. (Ed.), *Adult Learning Methods: A Guide for Effective Instruction* (Kreiger, Melbourne, 1989).
17. Bergevin, P., Morris, D. and Smith, R. M., *Adult Education Procedures: A Handbook of Tested Patterns for Effective Particpation* (The Seabury Press, 1963).
18. Bradley, D. L., *Perspectives on Adult Learning,* Ed. Brady, E. M. (College of Education Publishers, University of Southern Maine, 1986).
19. Deshler, D. (Ed.), *New Directions for Continuing Education* (Jossey-Bass, San Francisco, 1984).

